# Prediction of major adverse cardiac events in the emergency department using an artificial neural network with a systematic grid search

**DOI:** 10.1186/s12245-023-00573-2

**Published:** 2024-01-04

**Authors:** Ahmed Raheem, Shahan Waheed, Musa Karim, Nadeem Ullah Khan, Rida Jawed

**Affiliations:** 1 Department of Emergency Medicine, Aga Khan University Hospital, Karachi, Pakistan; 2 Department of Clinical Research, National Institute of Cardiovascular Diseases (NICVD), Karachi, Pakistan

**Keywords:** Artificial intelligence, Emergency medicine, Cardiac arrest, Major adverse cardiovascular events, Validation study

## Abstract

**Background:**

The aim of our research was to design and evaluate an Artificial Neural Network (ANN) model using a systemic grid search for the early prediction of major adverse cardiac events (MACE) among patients presenting to the triage of an emergency department.

**Methods:**

This is a single-center, cross-sectional study using electronic health records from January 2017 to December 2020. The research population consists of adults coming to our emergency department triage at Aga Khan University Hospital. The MACE during hospitalization was the main outcome. To enhance the architecture of an ANN using triage data, we used a systematic grid search strategy. Four hidden ANN layers were used, followed by an output layer. Following each hidden layer was back normalization and a dropout layer. MACE was predicted using three binary classifiers: ANN, Random Forests (RF), and logistic regression (LR). The overall accuracy, sensitivity, specificity, precision, and recall of these models were examined. Each model was evaluated using the receiver operating characteristic curve (ROC) and an F1-score with a 95% confidence interval.

**Results:**

A total of 97,333 emergency department visits were recorded during the study period, with 33% of patients having cardiovascular symptoms. The mean age was 54.08 (19.18) years old. The MACE was observed in 23,052 (23.7%) of the patients, in-hospital (up to 30 days) mortality in 10,888 (11.2%) patients, and cardiac arrest in 5483 (5.6%) patients. The data used for training and validation were 77,866 and 19,467 in an 80:20 ratio, respectively. The AUC score for MACE with ANN was 0.97, which was greater than RF (0.96) and LR (0.96). Similarly, the precision-recall curve for MACE utilizing ANN was greater (0.94 vs. 0.93 for RF and 0.93 for LR). The sensitivity for MACE prediction using ANN, RF, and LR classifiers was 99.3%, 99.4%, and 99.2%, respectively, with the specificities being 94.5%, 94.2%, and 94.2%, respectively.

**Conclusion:**

When triage data is used to predict MACE, death, and cardiac arrest, ANN with systemic grid search gives precise and valid outcomes and will benefit in predicting MACE in emergency rooms with limited resources that have to deal with a substantial number of patients.

**Supplementary Information:**

The online version contains supplementary material available at 10.1186/s12245-023-00573-2.

## Introduction

Cardiovascular disease is a major cause of illness and death worldwide [1], with the presentation of acute chest discomfort in five to twenty percent of emergency room visits [2–5], ranging from benign to potentially fatal, such as aortic dissection, pulmonary embolism, and acute coronary syndrome [6, 7].

In a busy emergency setting, it is difficult to classify and treat the subgroups of people with cardiovascular disease; hence, prediction scores such as HEART [7, 8], GRACE (Global Registry of Acute Coronary Events) [9], and TIMI (thrombolysis in myocardial infarction) [10], have emerged to facilitate the process to aid in disposition and decision-making. However, in overcrowded and busy emergency rooms, the time required to utilize these scoring systems might be troublesome [11]. Hence, in recent years, artificial intelligence (AI) and machine learning (ML) applications in emergency care have emerged [12], with studies showing that ML outperforms conventional measures by managing the many factors accessible via electronic medical records and big data [13–15]. Many different studies employed AI algorithms to generate scores for mortality interpretation as a method for establishing triage ordering [16–20].

For evaluations of the major adverse cardiac effect (MACE), previous studies have included regression-based models such as the Framingham Risk Score (FRS), the GRACE, and the TIMI research [21]. However, these models do not consider the intricate relationships between clinical factors. The systemic grid search approach is deemed superior for multivariable, complicated multiple logistic regression (LR) and, simpler to execute with non-functional data and has shown high performance based on clinical presentation parameters [22]. Also, the evaluation of predictor variables using various machine learning algorithms and systemic grid approaches serves well in the analysis of non-functional data with hyperparameters.

An artificial neural network (ANN) emulates the structural framework of biological neural networks. Comprising interconnected artificial neurons arranged in layers, this computational model undergoes a training process to optimize the weights of connections. Through this refinement, the algorithm acquires the capability to discern patterns, relationships, and features within data, facilitating accurate predictions and classifications on novel, unseen datasets. Therefore, in our study, we aim to develop and validate an ANN model and utilize the systemic grid search based on triage presentation vitals and cardiovascular symptoms to predict MACE at the triage level in an emergency setting. This approach shows intricate connections among factors and produces reliable, predictive forecasts for cardiac outcomes. Hence, our model will help prevent MACE by allowing early detection and timely intervention in the emergency department.

## Methods

### Study design, setting, and population

This was a cross-sectional study undertaken in the emergency department at Aga Khan Hospital, which is an urban, 62-bed emergency department that serves almost 60,000 patients yearly. The triage data utilized for this research was gathered from hospital electronic databases from December 2017 to December 2020. All individuals (age 18 and older) who arrived at the emergency room with cardiovascular symptoms were included in the research. In the index ED visit, the criteria for cardiovascular symptoms (chest pain, sweating, shortness of breath, shoulder pain, arm pain, jaw pain, impending doom, hypotension, etc.) were derived according to the International Classification of Diseases, Tenth Revision, Chapter 11 (ICD-10) [23]. Excluded were patients who lacked a disposition record, were deceased upon admission, were moved to another hospital from the ED, or were discharged against medical advice (LAMA).

**Fig. 1:**
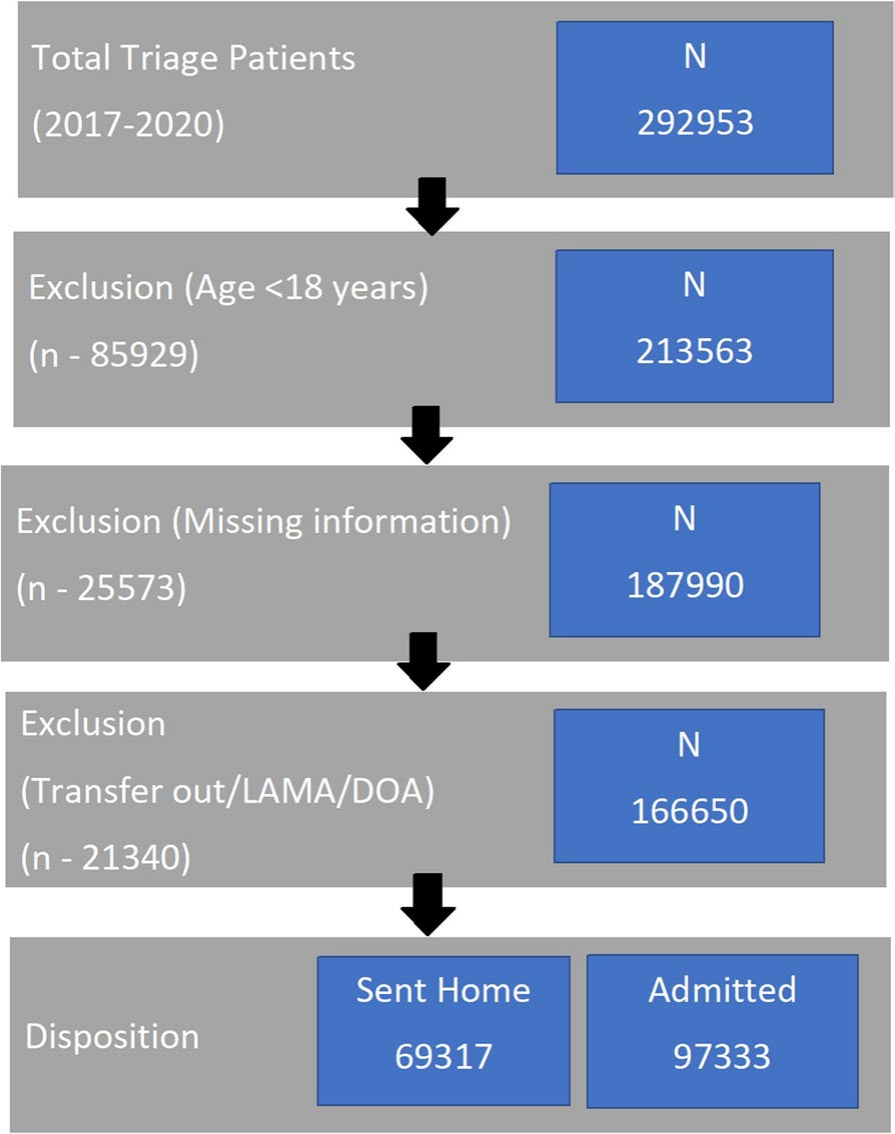
Flow chart showing the exclusion criteria and final number of patients included in the analysis

Between January 1, 2017, and December 31, 2020, a total of 292,953 patients presented to the triage of the emergency department, where the emergency severity index (ESI), a five-level scale, was used to triage the patients [24]. Eighty-five thousand nine hundred twenty-nine patients who were less than 18 years old were removed, while 25,573 were eliminated owing to missing information, which included missing hospital registration numbers as well as missing outcome information. In day-to-day emergency services, some patients reach the triage counter but are not admitted into the emergency room due to either overcrowding (diversion) or being left without being seen (LWBS). Further, 21,440 patients were excluded due to transfer out, LAMA (Leaving Against Medical Advice), and death on arrival (DOA). The remaining 69,317 were sent home, and the actual sample size was 97,333 (Fig. 1).

The sample size represented all individuals who presented to the triage of our emergency department with cardiovascular-related complaints. According to the ESI triage categorization, the patients were further subdivided into high-risk (P1 and P2) and low-risk (P3, P4, and P5). For reporting observational research, the STROBE (Strengthening the Reporting of Observational Studies in Epidemiology) checklist for observational studies was used.

### Data features and missing data imputation

The dataset comprised 44 feature variables of demographic and clinical characteristics of patients, among which several were predictors of MACE, mortality, and cardiac arrest based on prior research. The variables included age (years), gender, triage vitals (systolic blood pressure, diastolic blood pressure, respiratory rate, oxygen saturation, and temperature), presenting symptoms, cardiac diagnosis, pre-existing comorbidities, triage category, triage processing time windows, and disposition, as well as the outcome variable of all-cause in-hospital (up to 30 days) mortality, cardiac arrest, and MACE (STEMI (ST-segment elevation myocardial infarction), NSTEMI (non-ST elevation myocardial infarction), acute pulmonary edema, heart failure, and cardiogenic shock). Electrocardiography was not used owing to its technical limitations and interpretive variability. Our computerized triage record had 2.6% missing triage vitals and 0.6% missing category covariates, which were subsequently accounted for using the imputation method based on random forests (RF) algorithms. The imputation algorithm was initiated with the average values for continuous variables and the most frequent category for the categorical variables.

### Outcome measurements

We established MACE (STEMI, NSTEMI, acute pulmonary edema, heart failure, and cardiogenic shock) during the hospital stay as primary outcome metrics for our investigation. The secondary outcomes were all-cause in-hospital (up to 30 days) and cardiac arrest following the initial ED visit.

### Data processing and model development

In the absence of an independent study cohort for the validation of models, we have adopted the train-test split approach to develop and test the prediction accuracy of the models. The complete dataset was split into two sets: the training dataset, which consisted of 80% randomly selected cases, and the testing dataset, which consisted of the remaining 20% of random observations (holdout cases). The training dataset was used to train the model for outcome prediction, and the testing dataset was used to assess the performance of the trained model for outcome prediction. Three binary classifiers, ANN, RF, and LR, were trained and evaluated for three distinct tasks, namely the prediction of MACE, in-hospital (up to 30 days) mortality, and cardiac arrest.

### ANN classifier

The optimal hyperparameter setting and structure of the ANN model were identified with the help of a grid search strategy. Based on the classifier’s performance, a model with four hidden layers was used. Grid search was used to adjust a total of nine hyperparameters of the ANN model, including the number of neurons in the first hidden layer [search space 1000, 800, 600], the number of neurons in the second hidden layer [search space 600, 400, 200], the number of neurons in the third hidden layer [search space 400, 200, 100], the number of neurons in the fourth hidden layer [search space 100, 50, 25], the learning rate [search space 1e − 1, 1e − 2, 1e − 3, 1e − 4, 1e − 5], the dropout rate [search space 0.4, 0.5, 0.6, 0.7], activation function “LeakyReLU” parameter alfa [search space 0.01, 0.02, 0.03, 0.04, 0.05], batch size for batch normalization [search space 8, 16, 32, 64], and number of epochs [search space 10, 15, 20, 30]. Each hidden layer was followed by a dropout layer and batch normalization. The activation function was “LeakyReLU” with “binary crossentropy” loss function. Using a grid search strategy, the ideal hyperparameters’ settings and training parameters for the three tasks namely the prediction of MACE, cardiac arrest, and in-hospital (up to 30 days) mortality.

### RF classifier

In the same way, three different RF classifiers were trained and tested to see how well they could predict MACE, in-hospital (up to 30 days) mortality, and cardiac arrest. The grid search approach was adopted to optimize the four parameters of the RF classifier. The four hyperparameters and their corresponding search spaces are as follows: the number of trees [search space 100, 200, 500], the size of the random subsets of features to consider when splitting a node [search space: auto,' sqrt, log2, and the search space for the random subsets of features], the depth of each tree in the forest [search space 6, 7, 8, 9, 10], and the criteria for splitting nodes in a decision tree [search space: ‘Gini,’ ‘entropy’].

### LR classifier

Finally, classifiers based on binary LR were developed to predict the three outcome variables, namely MACE, in-hospital (up to 30 days) mortality, and cardiac arrest.

In addition to comparing ANN, RF, and LR with one another for predicting mortality, cardiac arrest, and MACE, the Emergency Severity Index (ESI), a routinely used risk stratification modality in the ED, was compared with the three classifiers. The prediction performance of each of the classifiers and the ESI was evaluated using the validation dataset.

In the validation dataset, all models were tested for their overall accuracy of prediction. The sensitivity, specificity, recall precision, and F1-score of each model were calculated. Using the predicted probabilities of ANN, RF, and LR classifiers, the receiver operating characteristic (ROC) curve analysis was performed, and the area under the curve (AUC) was evaluated across these three outcomes.

### Feature selection

Using the “featurewiz” technique, significant features for the classification of three outcome variables, namely in-hospital (up to 30 days) mortality, cardiac arrest, and MACE, were obtained separately. In supplemental files, the specifics of the chosen characteristics for each of the three jobs are described (Table S1 and Table S2). The prediction performance of three classifiers was examined using both whole and feature-selected datasets.

## Results

A total of 97,333 patients were included in our study, and 33.3% of individuals had cardiovascular symptoms. The mean age of patients was 54.08 years (SD 19.18), and 5170 (53.0%) were male (Table 1). The average ED admission time was 7.87 h (SD 4.37), and the average ED departure time was 8.97 h (SD 4.37). The distribution of the ESI levels was as follows: P1 30,037 (30.9%), P2 33,986 (34.9%), P3 30,411 (31.2%), P4 1688 (1.7%), and P5 1211 (1.2%). According to risk classification, 64,043 or 65.8% were classified as high-risk. At the time of the patient’s presentation in the ED, shortness of breath was the most prevalent presenting symptom noticed in 22,881 cases (23.5%).

**Table 1:** Comparison of training and validation dataset and outcomes of emergency triage dataset (*n* = 97,333)

	Training data	Validation data	Total
	[*n* = 77,866]	[*n* = 19,467]	[*n* = 97,333]
Age in (years)	54.04 (± 19.20)	54.23 (± 19.11)	54.08 (± 19.18)
ED triage to admission time	7.87 (± 4.37)	7.86 (± 4.36)	7.87 (± 4.37)
ED triage to ED discharge time	8.97 (± 4.38)	8.96 (± 4.36)	8.97 (± 4.37)
Triage vitals			
SBP (mmHg)	133.45 (± 21.97)	133.32 (± 21.9)	133.43 (± 21.95)
DBP (mmHg)	76.86 (± 14.36)	76.61 (± 14.38)	76.81 (± 14.36)
RR (breaths/min)	24.58 (± 7.87)	24.65 (± 7.9)	24.59 (± 7.88)
Temperature (0C)	37.32 (± 0.78)	37.32 (± 0.77)	37.32 (± 0.78)
Oxygen saturation (%)	94.46 (± 7.46)	93.95 (± 6.56)	94.44 (± 5.48)
Heart rate (BPM)	102.18 (± 23.13)	102 (± 23.08)	102.14 (± 23.12)
Gender			
Male	41,402 (53.2%)	10,298 (52.9%)	51,700 (53.1%)
Female	36,464 (46.8%)	9169 (47.1%)	45,633 (46.9%)
Triage category			
P1	23,968 (30.8%)	6069 (31.2%)	30,037 (30.9%)
P2	27,257 (35%)	6729 (34.6%)	33,986 (34.9%)
P3	24,331 (31.2%)	6080 (31.2%)	30,411 (31.2%)
P4	1337 (1.7%)	351 (1.8%)	1688 (1.7%)
P5	973 (1.2%)	238 (1.2%)	1211 (1.2%)
Risk stratification			
Low risk (P1 and P2)	26,624 (34.2%)	6666 (34.2%)	33,290 (34.2%)
High risk (P3, P4, and P5)	51,242 (65.8%)	12,801 (65.8%)	64,043 (65.8%)
Seasons			
Winter (December, January, and February)	27,650 (35.5%)	7029 (36.1%)	34,679 (35.6%)
Summer (April, May, and June)	30,881 (39.7%)	7628 (39.2%)	38,509 (39.6%)
Weeks			
Weekend	22,783 (29.3%)	5657 (29.1%)	28,440 (29.2%)
Weekday	55,083 (70.7%)	13,810 (70.9%)	68,893 (70.8%)
Comorbid			
Diabetes mellitus	14,732 (18.9%)	3727 (19.1%)	18,459 (19%)
Hypertension	18,363 (23.6%)	4648 (23.9%)	23,011 (23.6%)
Ischemic heart disease	4559 (5.9%)	1118 (5.7%)	5677 (5.8%)
Triage presenting complaints/diagnosis			
Shortness of breath	18,298 (23.5%)	4583 (23.5%)	22,881 (23.5%)
Sepsis	3383 (4.3%)	844 (4.3%)	4227 (4.3%)
Chest pain	6786 (8.7%)	1689 (8.7%)	8475 (8.7%)
Sweating	535 (0.7%)	124 (0.6%)	659 (0.7%)
Tachycardia	1546 (2%)	397 (2%)	1943 (2%)
Weakness	6640 (8.5%)	1678 (8.6%)	8318 (8.5%)
COVID-19	4413 (5.7%)	1100 (5.7%)	5513 (5.7%)
Shoulder pain	4 (0%)	3 (0%)	7 (0%)
Epigastric pain	113 (0.1%)	29 (0.1%)	142 (0.1%)
Cardiac parameters			
Complete heart block	127 (0.2%)	45 (0.2%)	172 (0.2%)
STEMI	4946 (6.4%)	1283 (6.6%)	6229 (6.4%)
NSTEMI	4544 (5.8%)	1083 (5.6%)	5627 (5.8%)
Unstable angina	142 (0.2%)	38 (0.2%)	180 (0.2%)
Heart failure	934 (1.2%)	237 (1.2%)	1171 (1.2%)
Cardiogenic shock	3834 (4.9%)	976 (5%)	4810 (4.9%)
Syncope	336 (0.4%)	88 (0.5%)	424 (0.4%)
Atrial fibrillation	613 (0.8%)	169 (0.9%)	782 (0.8%)
Acute pulmonary edema	1600 (2.1%)	393 (2%)	1993 (2%)
Fluid overload	1402 (1.8%)	381 (2%)	1783 (1.8%)
Hypertrophy	7 (0%)	3 (0%)	10 (0%)
Cardiac tamponade	10 (0%)	2 (0%)	12 (0%)
Mortality			
Alive	69,191 (88.9%)	17,254 (88.6%)	86,445 (88.8%)
Death	8675 (11.1%)	2213 (11.4%)	10,888 (11.2%)
Cardiac arrest	4396 (5.6%)	1087 (5.6%)	5483 (5.6%)

The MACE was observed in 23,052 (23.7%) of the patients, In-hospital (up to 30 days) mortality in 10,888 (11.2%) patients, and cardiac arrest in 5483 (5.6%) patients.

**Fig. 2:**
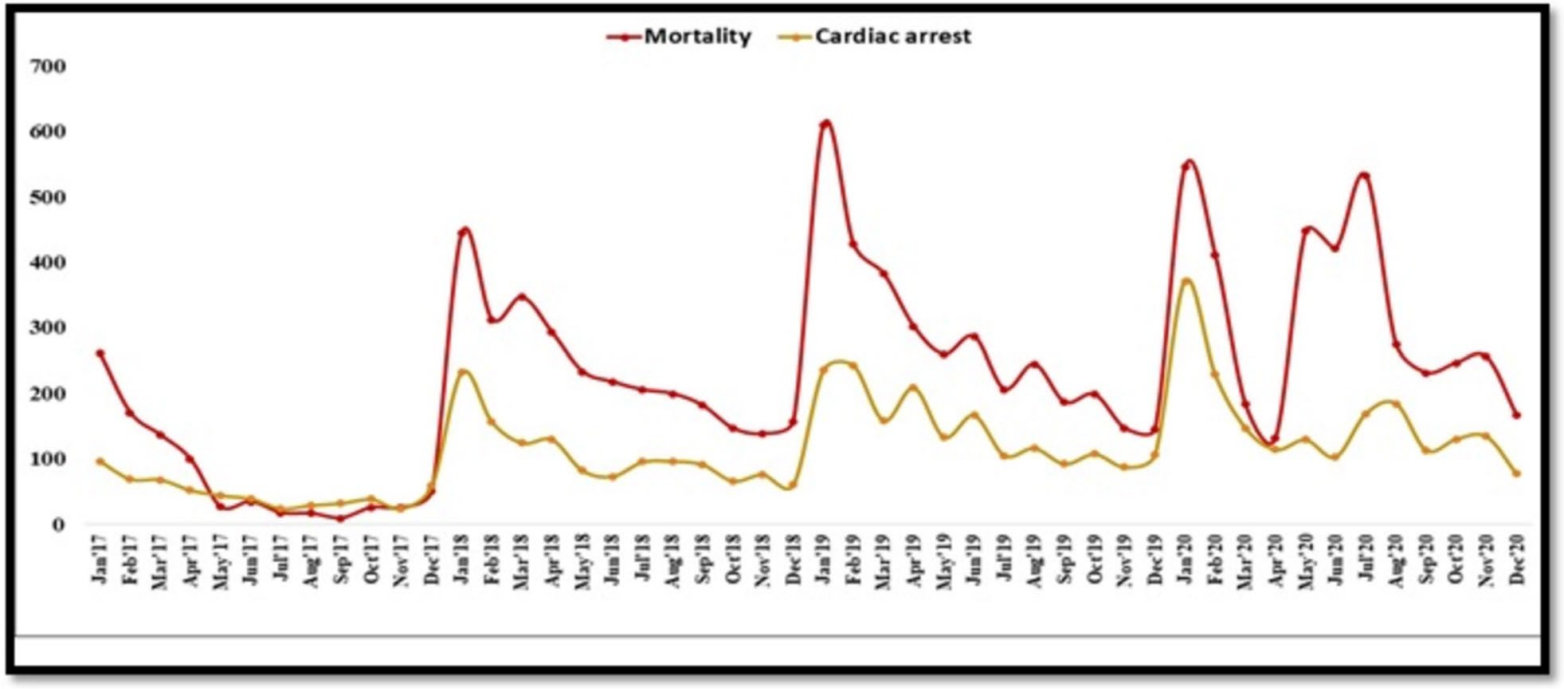
Seasonal variation of the number of cases of mortality and cardiac arrest

The distribution of diagnoses revealed that the majority of patients had STEMI (6229), followed by NSTEMI (5627), heart failure (1171), cardiogenic shock (4810), and acute pulmonary edema (1783). Seasonal variation in mortality and cardiac arrest was observed in our data (Fig. 2). 34,679 (35.6%) patients presented in winter, i.e., December, January, and February, whereas 38,509 (39.5%) presented in summer, i.e., April, May, and June of 2020. The majority of patients, around 68,893 (70.8%) presented on weekdays.

The proposed ANN structure sequential model of implementation of the best selected ANN model architecture for in-hospital (up to 30 days) mortality, cardiac arrest, and MACE was 491,801, 353,951, and 178,501, respectively, as trainable parameters. Trainable parameters distributed by layers are presented in Additional file 1.

**Fig. 3:**
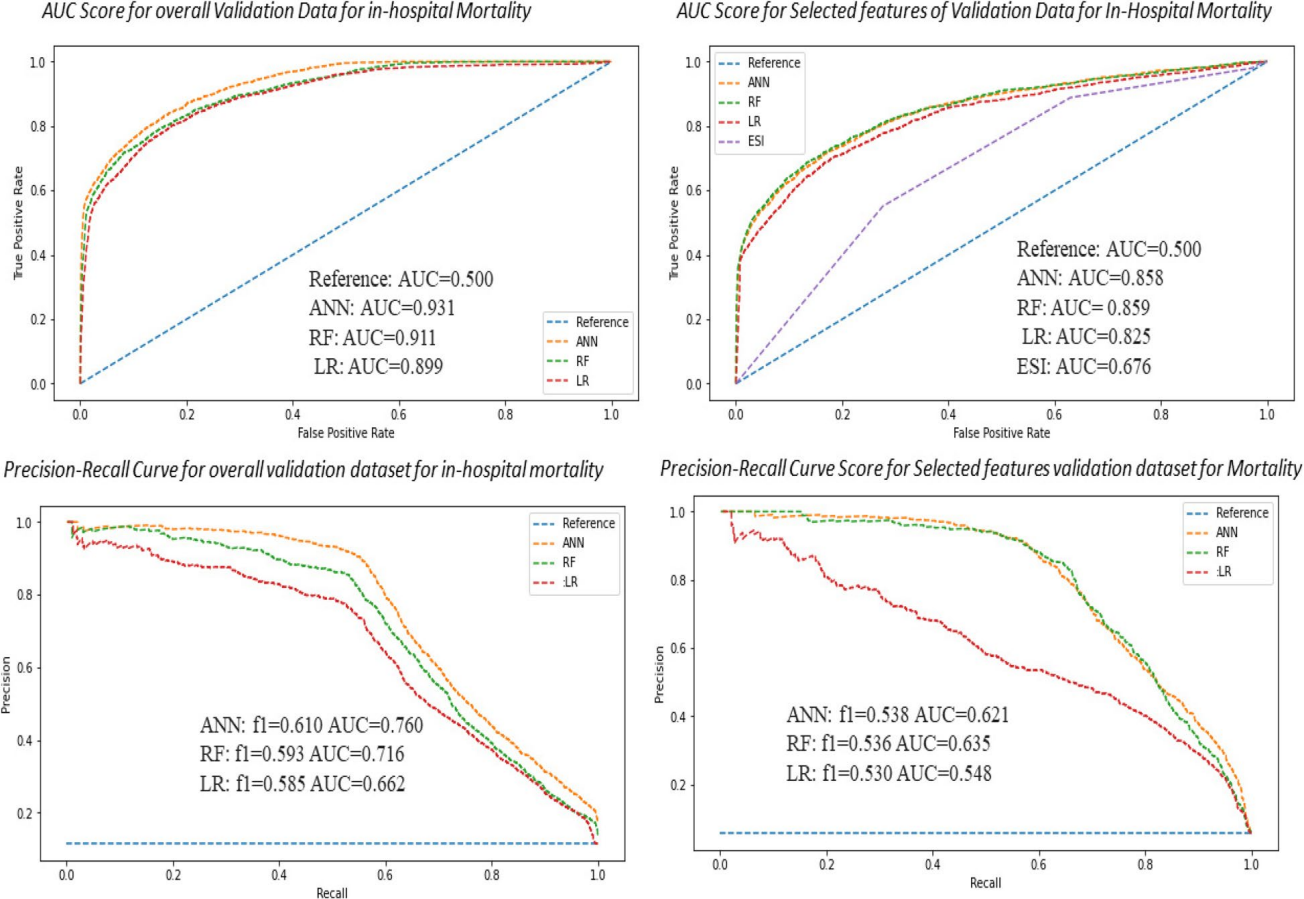
Precision-recall curve and AUC curve for artificial neural network (ANN) to other models comparison with RF and LR analysis that predicts 30-day hospital mortality with and without selected features

**Fig. 4:**
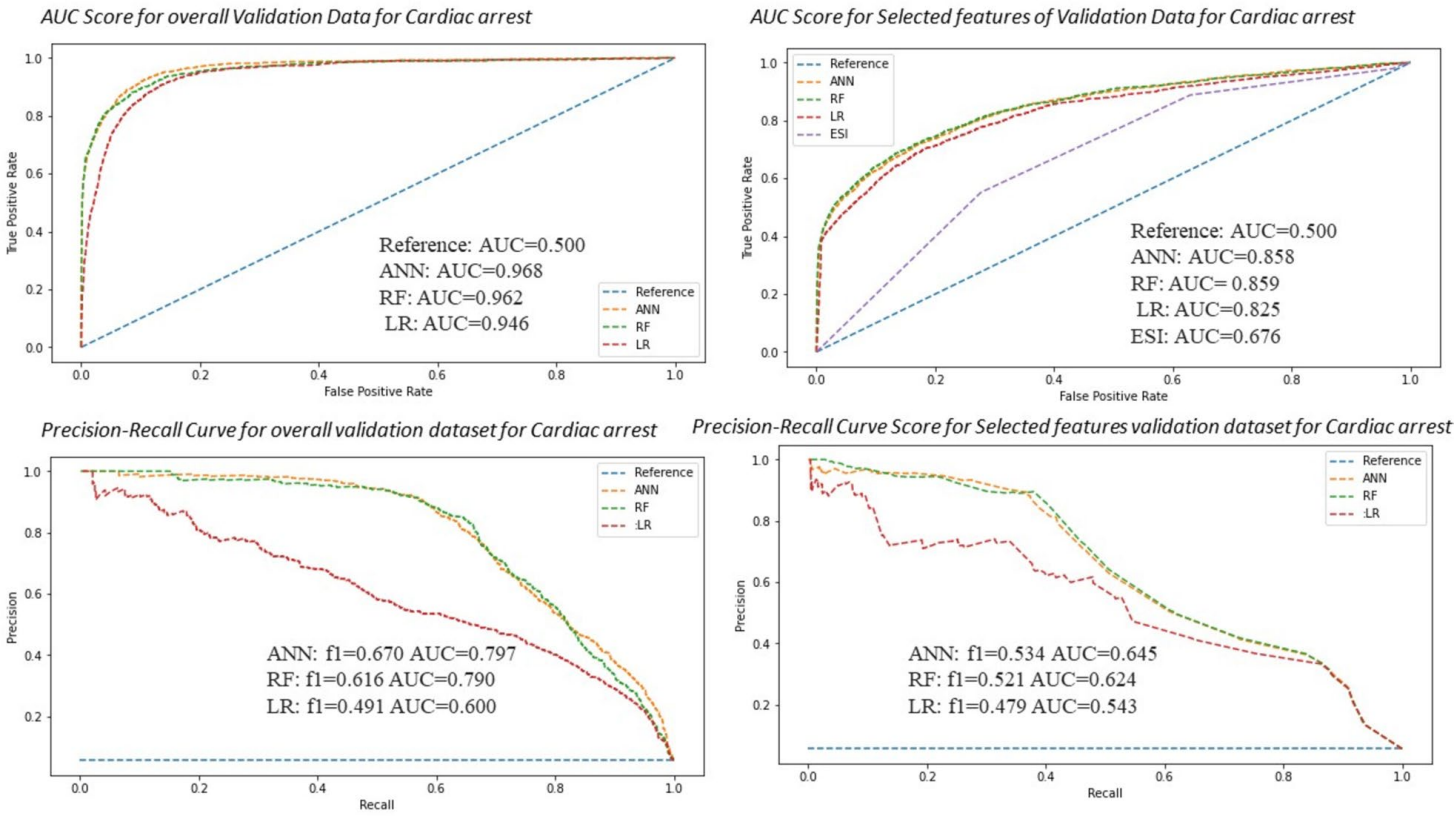
Precision-recall curve and AUC curve for artificial neural network (ANN) to other models comparison with RF and LR analysis that predicts cardiac arrest in ED with and without selected features

**Fig. 5:**
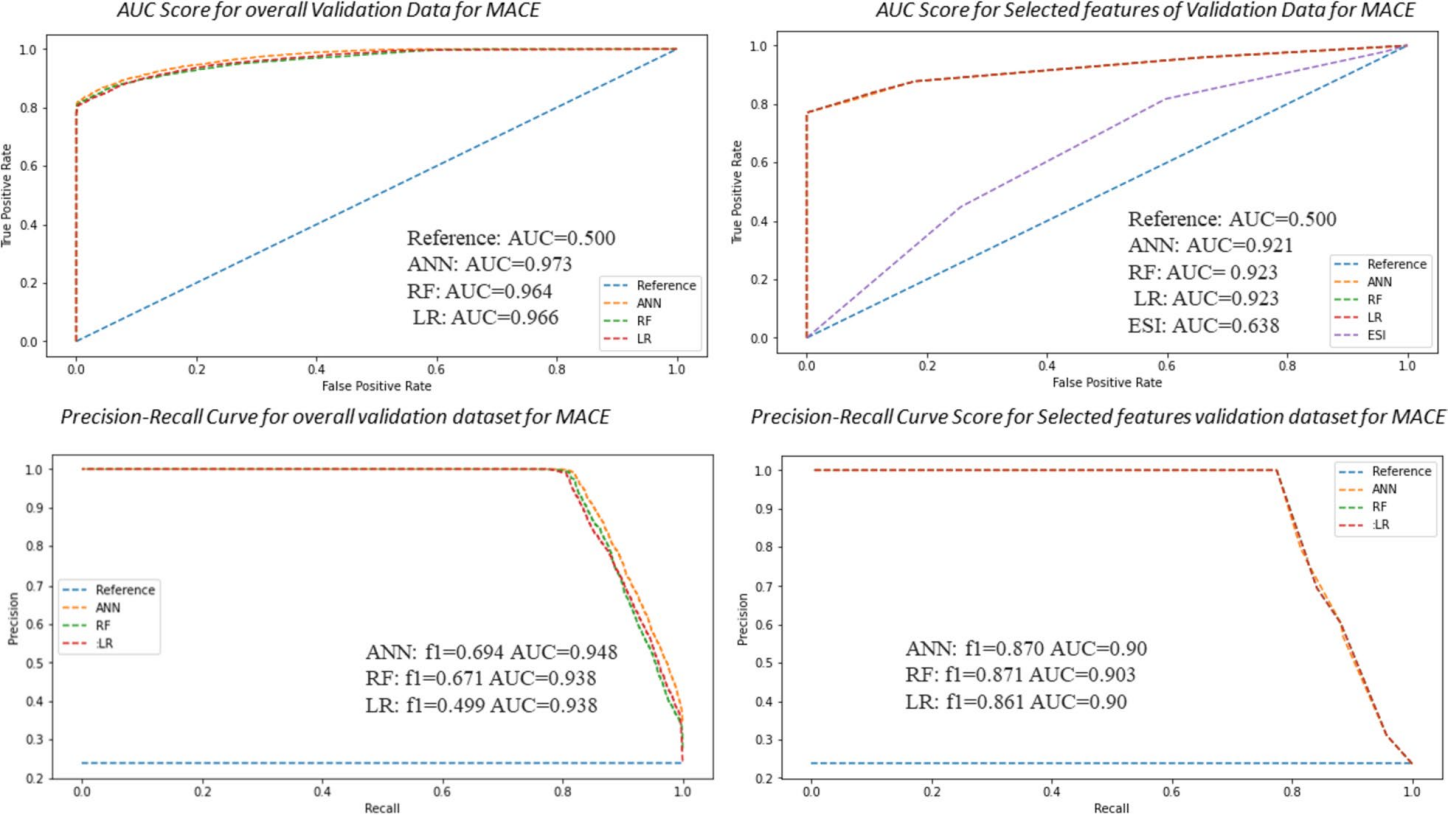
Precision-recall curve and AUC curve for artificial neural network (ANN) to other models comparison with RF and LR analysis that predicts MACE in ED with and without selected features

The AUC in the validation dataset was 0.931 for the ANN, 0.911 for the RF, and 0.889 for the LR classifier for predicting in-hospital mortality, with f1-scores of 0.610, 0.593, and 0.585, respectively (Fig. 3). Similarly, the AUC in the validation dataset for cardiac arrest was 0.968 for ANN, 0.962 for RF, and 0.946 for the LR classifier, with f1-scores of 0.67, 0.61, and 0.49, respectively (Fig. 4). In addition, the prediction of MACE and AUC in the validation data was 0.973 for ANN, 0.964 for RF, and 0.966 for LR, with f1-scores of 0.694, 0.671, and 0.499, respectively (Fig. 5). The AUCs of various models were compared to see the significant differences (Table 2).

**Table 2:** Comparison of AUC of different models

	DeLong test to compare AUCs for validation dataset with selected features					
	ANN vs. ESI	ANN vs. RF	ANN vs. Logistic	RF vs. ESI	Logistic vs. ESI	RF vs. logistic
In-hospital mortality	< 0.001*	0.486	0.325	< 0.001*	< 0.001*	0.045*
Cardiac arrest	< 0.001*	0.589	0.151	< 0.001*	< 0.001*	0.645
MACE	< 0.001*	0.638	0.956	< 0.001*	< 0.001*	0.185

*AUC* area under curve, *ANN* artificial neural network, *MACE* major adverse cardiac event, *ESI* emergency severity index, *RF* random forests, *LR* logistic regression

*Significant

The sensitivity for the prediction of in-hospital mortality was 94.6%, 87.9%, and 79.7% for the ANN, RF, and LR classifiers, with specificities of 93.3%, 93.3%, and 93.4%, respectively. Similarly, the sensitivity for cardiac arrest prediction using ANN, RF, and LR classifiers was 93.4%, 94.8%, and 68.5%, with specificities of 97.2%, 96.8%, and 96.4%, respectively. Furthermore, the sensitivity for MACE prediction using ANN, RF, and LR classifiers was 99.3%, 99.4%, and 99.2%, respectively, with the specificities being 94.5%, 94.2%, and 94.2%, respectively (Table 3).

**Table 3:** Sensitivity and specificity analysis of the reference and machine learning models in the overall and selected validation set

	Validation			Selected features for validation		
	Acc	Sens	Spec	Acc	Sens	Spec
	(95% CI)	(95% CI)	(95% CI)	(95% CI)	(95% CI)	(95% CI)
In-hospital mortality						
ANN classifier	93.40%	94.60%	93.30%	92.46%	85.43%	92.84%
	(93.1% to 93.8%)	(93.1% to 95.9%)	(93.02% to 93.7%)	(92.08% to 92.83%)	(83.09% to 87.56%)	(92.46% to 93.21%)
RF classifier	93.02%	87.90%	93.30%	92.52%	87.53%	92.78%
	(92.6% to 93.3%)	(85.9% to 89.8%)	(92.9% to 93.6%)	(92.14% to 92.89%)	(85.27% to 89.55%)	(92.40% to 93.15%)
LR	92.50%	79.70%	93.40%	92.36%	84.93%	92.76%
	(92.1% to 92.9%)	(77.4% to 81.9%)	(93.09% to 93.8%)	(91.98% to 92.73%)	(82.55% to 87.11%)	(92.38% to 93.13%)
Cardiac arrest						
ANN classifier	97.10%	93.40%	97.20%	95.93%	86.40%	96.14%
	(96.8% to 97.3%)	(91.15% to 95.2%)	(97.0% to 97.4%)	(95.64% to 96.20%)	(82.74% to 89.53%)	(95.86% to 96.41%)
RF classifier	96.80%	94.80%	96.80%	96.02%	87.82%	96.21%
	(96.5% to 97.0%)	(92.5% to 96.5%)	(96.6% to 97.1%)	(95.74% to 96.29%)	(84.34% to 90.77%)	(95.93% to 96.47%)
LR	95.50%	68.50%	96.40%	95.10%	61.04%	96.28%
	(95.2% to 95.8%)	(64.6% to 72.2%)	(96.1% to 96.7%)	(94.79% to 95.40%)	(57.18% to 64.81%)	(96.00% to 96.55%)
Major adverse cardiac events (MACE)						
ANN classifier	95.40%	99.30%	94.50%	99.73%	93.35%	93.50%
	(95.1% to 95.7%)	(99.06% to 99.5%)	(94.1% to 94.9%)	(98.53% to 99.99%)	(92.95% to 93.73%)	(93.11% to 93.87%)
RF classifier	95.20%	99.40%	94.20%	99.97%	93.35%	94.56%
	(94.9% to 95.5%)	(99.2% to 99.6%)	(93.8% to 94.6%)	(99.84% to 100.00%)	(92.95% to 93.73%)	(94.23% to 94.87%)
LR	95.20%	99.02%	94.20%	99.78%	93.35%	94.52%
	(94.8% to 95.5%)	(98.6% to 99.3%)	(93.9% to 94.6%)	(99.56% to 99.90%)	(92.95% to 93.73%)	(94.20% to 94.84%)

*Acc*. accuracy, *Sens*. sensitivity, *Spec.* specificity, *ANN* artificial neural network, *RF* random forests, *LR* logistic regression

## Discussion

In this retrospective, single-center, cross-sectional study, we describe a new, more accurate way to predict MACE, in-hospital (up to 30 days) mortality from all causes, and cardiac arrest using a systemic grid technique in an ANN. The extensive dataset, comprising 97,333 patients, allowed for a robust analysis of the performance of the proposed ANN model in comparison to RF and LR classifiers and routinely used emergency severity index (ESI).

Of the three models we have chosen, ANN has an AUC of 0.97 for MACE, 0.968 for cardiac arrest, and 0.931 for in-hospital (up to 30 days) mortality. The implementation of an ANN model for early prediction of MACE at the ED triage has the potential to revolutionize current practices and significantly improve patient outcomes. The systemic grid search approach aims to enhance the model's performance, ensuring optimal predictive capabilities. Comparison with traditional risk assessment methods will provide insights into the added value of the proposed model in the emergency care setting. By manipulating the hyperparameters of the various models, ANN with a systemic grid search approach provided the highest accuracy among the different models (RF and LR). The integration of machine learning models into routine ED procedures presents challenges related to interpretability, scalability, and real-time applicability. Addressing these challenges is crucial for the successful implementation of such models in clinical practice [25].

The use of artificial intelligence for the prediction of various outcomes in the emergency department has gained popularity among researchers in recent years. Jang DH et al. [26] developed and evaluated ANN classifiers for early detection of patients at risk of cardiac arrest in overcrowded emergency departments. The research utilized a single-center electronic health record (EHR)-based approach and compared three ANN models (multilayer perceptron-MLP, long-short-term memory-LSTM, and hybrid) with other classifiers such as the modified early warning score (MEWS), logistic regression, and random forest. In a dataset of 374,605 emergency department visits, the ANN models consistently outperformed non-ANN models. The area under the receiver operating characteristic curve (AUROC) values for ANN models (MLP 0.929, LSTM 0.933, and hybrid 0.936) surpassed those of non-ANN models, with the hybrid model demonstrating the highest performance. Similar to our findings, ANN classifiers exhibited superior test characteristics, including sensitivity, specificity, positive predictive value (PPV), and negative predictive value (NPV), particularly when compared with MEWS thresholds and each other.

The study by Wu CC et al. [27] addresses the lack of risk scores to distinguish non-ST-elevation myocardial infarction (NSTEMI) from non-cardiogenic chest pain, aiming to reduce misdiagnosis in emergency departments. Employing an artificial intelligence (AI) approach, an ANN model was developed using data from 268 chest pain patients. The model demonstrated high accuracy (92.86%) and an impressive AUC of 98.4%. The ANN model exhibited strong sensitivity (90.91%), specificity (93.33%), positive predictive value (76.92%), and negative predictive value (97.67%).

Another study by Hong WS et al. [28] aimed to predict hospital admission at ED triage by incorporating patient history alongside triage information. In a retrospective analysis of 560,486 adult ED visits, three types of classifiers (logistic regression, gradient boosting, and deep neural networks) were trained on datasets containing triage information, patient history, and the full set of variables. The models demonstrated robust predictive capabilities, with the inclusion of patient history significantly enhancing performance compared to triage information alone. The low-dimensional XGBoost model, utilizing variables such as ESI level, outpatient medication counts, demographics, and hospital usage statistics, achieved an impressive AUC of 0.91. The findings underscore the effectiveness of machine learning in predicting hospital admission, emphasizing the importance of incorporating patient history for improved accuracy in admission risk assessment during ED triage.

Similarly, Goto T, et al. [29] have recently assessed the performance of machine learning approaches in predicting clinical outcomes and disposition for children in ED triage, comparing them with conventional triage methods. The study focused on critical care (admission to ICU and/or in-hospital death) and hospitalization outcomes. Machine learning models (lasso regression, RF, gradient-boosted decision tree, and deep neural network) were developed using routinely available triage data as predictors. Results showed that machine learning models, especially for hospitalization prediction, outperformed conventional triage methods, demonstrating higher discriminative ability and reducing both undertriage and overtriage of pediatric patients. The study concludes that machine learning-based triage could enhance prediction accuracy and improve patient disposition in pediatric emergency settings.

The significant cardiovascular burden of the South Asian population is reflected in our study’s prevalence of 33.3%, owing to a Mediterranean diet and a sedentary lifestyle [30]. The high prevalence of cardiovascular symptoms and better prediction of MACE, cardiac arrest, and in-hospital 30-day mortality by ANN provide significant evidence to incorporate this model in electronic triage systems at emergency departments.

Our data also contrasts with the emergency severity index (ESI), which is used to stratify patients at risk in routine clinical practice but has poor predictive capacity for identifying critically ill patients and a high degree of heterogeneity within each triage category [31]. Future research should compare this model to emergency physician gestalt in low-resource emergency rooms and assess patient outcomes.

## Limitations

Our research has several limitations. Firstly, due to the absence of external validation, our retrospective analysis employs data from a single institution, and hence, the performance of our model may not be generalizable. Secondly, ANN has issues with interpretability and inferences, may not operate with huge non-functional datasets, and requires a significant amount of time to generate findings. We have used the systematic grid search approach to solve this issue. However, we feel our method can accommodate hyperparameters given the retrospective nature of the data. Thirdly, we did not test the influence of our model on real-time data and clinical practice. This was beyond the scope of our research, but we will assess it in a future investigation. Fourthly, the potential for systemic bias in nurses’ practices is more prevalent in LMICs. There is an element of subjectivity in setting the triage ESI level, and any systemic bias would be mirrored in the model, preventing further generalizability. Our application of the ANN model in settings with limited resources will need an electronic health record with operational e-triage tools to make choices in real time. This is uncommon in many rural and some metropolitan places, which is also one of the weaknesses of our study. However, this research confirms the notion that LMICs should employ ANN as a support tool to aid doctors and reduce medical mistakes in their ED.

However, the application of artificial intelligence and machine learning in healthcare poses several difficulties, such as malpractice responsibility, patient satisfaction, insurance coverage, damage to physical integrity, innovation expenses, legal challenges, healthcare professional liability, and a dearth of high-quality data [32].

## Conclusion

For healthcare insurance in poor nations, a transparent and efficient data governance mechanism is essential, with technical and regulatory requirements supplemented using a humanistic-centered approach. ANN with systematic grid searching predicted MACE, cardiac arrest, and in-hospital 30-day mortality in triaging ED patients with cardiovascular symptoms with higher accuracy in contrast to LR and RF models. Our prediction model can, therefore, aid emergency room doctors in making prompt triage choices for patients with cardiovascular symptoms by categorizing and prioritizing patients in the early phase based on their triage presentation criteria.

### Supplementary Information

**Additional file 1: Table S1.** Significant features for the classification of three outcome variables.

**Additional file 2: Table S2.** Sensitivity & Specificity analysis of the reference and machine learning models in overall & selected training set.

## Data Availability

The dataset used and analyzed during the current study is available from the corresponding author on reasonable request.

## References

[1] Bayon Fernandez J, Alegria Ezquerra E, Bosch Genover X, Cabades OCA, Iglesias Garriz I, Jimenez Nacher J, et al. Grupo de Trabajo ad hoc de la Seccion de Cardiopatia Isquemica y Unidades Coronarias de la Sociedad Espanola de C. Chest pain units. Organization and protocol for the diagnosis of acute coronary syndromes. Rev Esp Cardiol. 2002;55(2):143–54. 10.1016/s0300-8932(02)76574-3 11852005

[2] Damman P, van’t Hof A, Ten Berg J, Jukema J, Appelman Y, Liem A, et al. 2015 ESC guidelines for the management of acute coronary syndromes in patients presenting without persistent ST-segment elevation: comments from the Dutch ACS working group. Netherlands Heart J. 2017;25(3):181–5. 10.1007/s12471-016-0939-y PMC5313450 27966184

[3] Members WC, Anderson JL, Adams CD, Antman EM, Bridges CR, Califf RM (2013). 2012 ACCF/AHA focused update incorporated into the ACCF/AHA 2007 guidelines for the management of patients with unstable angina/non–ST-elevation myocardial infarction: a report of the American College of Cardiology Foundation/American Heart Association Task Force on Practice Guidelines. Circulation.

[4] Niska R, Bhuiya F, Xu J. National Hospital Ambulatory Medical Care Survey: 2007 emergency department summary. Hyattsville: National Center for Health Statistics; 2010. 2012. 20726217

[5] Thygesen K, Alpert JS, Jaffe AS, Chaitman BR, Bax JJ, Morrow DA (2019). Fourth universal definition of myocardial infarction (2018). Eur Heart J.

[6] Lopez AD, Mathers CD, Ezzati M, Jamison DT, Murray CJ (2006). Global and regional burden of disease and risk factors, 2001: systematic analysis of population health data. Lancet.

[7] Rassi A, Rassi A, Little WC, Xavier SS, Rassi SG, Rassi AG (2006). Development and validation of a risk score for predicting death in Chagas' heart disease. N Engl J Med.

[8] DeLaney MC, Neth M, Thomas JJ (2017). Chest pain triage: Current trends in the emergency departments in the United States. J Nucl Cardiol.

[9] Brady W, de Souza K (2018). The HEART score: a guide to its application in the emergency department. Turkish J Emerg Med.

[10] Fox KA, Eagle KA, Gore JM, Steg PG, Anderson F, GRACE, et al. The global registry of acute coronary events, 1999 to 2009–GRACE. Heart. 2010;96(14):1095–101. 10.1136/hrt.2009.190827 20511625

[11] Backus BE, Six AJ, Kelder JC, Mast TP, van den Akker F, Mast EG (2010). Chest pain in the emergency room: a multicenter validation of the HEART Score. Crit Pathw Cardiol.

[12] Stewart J, Sprivulis P, Dwivedi G (2018). Artificial intelligence and machine learning in emergency medicine. Emerg Med Australas.

[13] Zhang P-I, Hsu C-C, Kao Y, Chen C-J, Kuo Y-W, Hsu S-L (2020). Real-time AI prediction for major adverse cardiac events in emergency department patients with chest pain. Scand J Trauma Resusc Emerg Med.

[14] Deo RC. Machine learning in medicine. Circulation [Internet]. 2015;132(20):1920–30. Available from: 10.1161/circulationaha.115.001593 10.1161/CIRCULATIONAHA.115.001593 PMC5831252 26572668

[15] Kakadiaris IA, Vrigkas M, Yen AA, Kuznetsova T, Budoff M, Naghavi M. Machine Learning Outperforms ACC/AHA CVD Risk Calculator in MESA. J Am Heart Assoc. 2018;7(22):e009476. 10.1161/JAHA.118.009476. 10.1161/JAHA.118.009476 PMC6404456 30571498

[16] Raita Y, Goto T, Faridi MK, Brown DF, Camargo CA, Hasegawa K (2019). Emergency department triage prediction of clinical outcomes using machine learning models. Crit Care.

[17] Zachariasse JM, Seiger N, Rood PP, Alves CF, Freitas P, Smit FJ, et al. Validity of the Manchester Triage System in emergency care: a prospective observational study. PLoS One. 2017;12(2). 10.1371/journal.pone.0170811 PMC5289484 28151987

[18] Levin S, Toerper M, Hamrock E, Hinson JS, Barnes S, Gardner H, et al. Machine-learning-based electronic triage more accurately differentiates patients with respect to clinical outcomes compared with the emergency severity index. Ann Emerg Med. 2018;71(5):565–74. e2. 10.1016/j.annemergmed.2017.08.005 28888332

[19] Christ M, Grossmann F, Winter D, Bingisser R, Platz E (2010). Modern triage in the emergency department. Dtsch Arztebl Int.

[20] Singh Y, Chauhan AS. Neural networks in data mining. J Theoretic Appl Inform Technol. 2009;5(1) 2018;7(22). Available from: 10.1161/jaha.118.009476.

[21] Antman EM, Cohen M, Bernink PJ, McCabe CH, Horacek T, Papuchis G (2000). The TIMI risk score for unstable angina/non–ST elevation MI: a method for prognostication and therapeutic decision making. JAMA.

[22] Pontes FJ, Amorim G, Balestrassi PP, Paiva A, Ferreira JR (2016). Design of experiments and focused grid search for neural network parameter optimization. Neurocomputing.

[23] So L, Evans D, Quan H (2006). ICD-10 coding algorithms for defining comorbidities of acute myocardial infarction. BMC Health Serv Res.

[24] Gilboy N, Tanabe P, Travers DA (2005). The Emergency Severity Index Version 4: changes to ESI level 1 and pediatric fever criteria. J Emerg Nurs.

[25] Fernandes M, Vieira SM, Leite F, Palos C, Finkelstein S, Sousa JM (2020). Clinical decision support systems for triage in the emergency department using intelligent systems: a review. Artif Intell Med.

[26] Jang DH, Kim J, Jo YH, Lee JH, Hwang JE, Park SM, Lee DK, Park I, Kim D, Chang H (2020). Developing neural network models for early detection of cardiac arrest in emergency department. Am J Emerg Med.

[27] Wu CC, Hsu WD, Islam MM, Poly TN, Yang HC, Nguyen PA, Wang YC, Li YC (2019). An artificial intelligence approach to early predict non-ST-elevation myocardial infarction patients with chest pain. Comput Methods Programs Biomed.

[28] Hong WS, Haimovich AD, Taylor RA (2018). Predicting hospital admission at emergency department triage using machine learning. PLoS One.

[29] Goto T, Camargo CA, Faridi MK, Freishtat RJ, Hasegawa K. Machine learning–based prediction of clinical outcomes for children during emergency department triage. JAMA Network Open. 2019;2(1):e186937-. 10.1001/jamanetworkopen.2018.6937 PMC6484561 30646206

[30] Jafary MH, Samad A, Ishaq M, Jawaid SA, Ahmad M, Vohra EA (2007). Profile of acute myocardial infarction (AMI) in Pakistan. Pakistan J Med Sci.

[31] Patel B, Sengupta P (2020). Machine learning for predicting cardiac events: what does the future hold?. Expert Rev Cardiovasc Ther.

[32] Sullivan HR, Schweikart SJ (2019). Are current tort liability doctrines adequate for addressing injury caused by AI?. AMA J Ethics.

