# Supplementary material for: Prediction of major adverse cardiac events in the emergency department using an artificial neural network with a systematic grid search
Source: Int J Emerg Med. 2024 Jan 4;17:4. doi: 10.1186/s12245-023-00573-2 (PMC10768150; doi:10.1186/s12245-023-00573-2)
Supplement: Supplementary file 1 — Additional file 1: Table S1. Significant features for the classification of three outcome variables. [file 12245_2023_573_MOESM1_ESM.docx]

**Features Selection for mortality**

['Cardiogenic_Shock', 'Dysphagia ', 'SOB', 'Warm_Season', 'IHD', 'Chest_Pain', 'HF', 'HTN', 'Sepsis', 'STEMI', 'SBP', 'Epigastric', 'RR', 'Synscope', 'Weekday', 'Temp', 'Reg_Admi_time', 'Tamponade', 'TIA', 'Weakness']

**Random Forest selected feature for Mortality**

RandomForestClassifier(max_features='auto', n_estimators= 200, max_depth=10, criterion='gini')

**Feature Selection for Cardiac Arrest**

['Dysphagia ', 'COVID_19', 'HTN', 'Techycardia', 'Cardiogenic_Shock', 'IHD', 'STEMI', 'TIA', 'Sepsis', 'Sweating']

**Random Forest selected feature for cardiac arrest**

{'criterion': 'gini', 'max_depth': 7, 'max_features': 'auto', 'n_estimators': 200}

**Feature Selection for MACE**

['STEMI', 'NSTEMI', 'HF', 'APE', 'Cardiogenic_Shock', 'Dysphagia', 'SOB', 'Warm_Season', 'Epigastric']

**Random Forest for mace with feature selection**

{'criterion': 'gini', 'max_depth': 7, 'max_features': 'auto', 'n_estimators': 100}

## Grid Search for hyperparameters of ANN model for selected feature for the prediction of In-Hospital Mortality

Model: "sequential"

_________________________________________________________________

Layer (type) Output Shape Param #

=================================================================

dense (Dense) (None, 400) 8400

dropout (Dropout) (None, 400) 0

dense_1 (Dense) (None, 200) 80200

batch_normalization (BatchN (None, 200) 800

ormalization)

dropout_1 (Dropout) (None, 200) 0

dense_2 (Dense) (None, 100) 20100

batch_normalization_1 (Batc (None, 100) 400

hNormalization)

dropout_2 (Dropout) (None, 100) 0

dense_3 (Dense) (None, 1) 101

=================================================================

Total params: 110,001

Trainable params: 109,401

Non-trainable params: 600

## Grid Search for hyperparameters of ANN model for selected feature for the prediction of cardiac arrest

[INFO] performing random search...

[INFO] best score is 0.96 using {'learnRate': 0.001, 'hiddenLayerTwo': 200, 'hiddenLayerThree': 100, 'hiddenLayerOne': 400, 'epochs': 20, 'dropout': 0.7, 'batch_size': 16, 'alfa': 0.04}

[INFO] evaluating the best model...

accuracy: 95.91%

Model: "sequential_1"

_________________________________________________________________

Layer (type) Output Shape Param #

=================================================================

dense_4 (Dense) (None, 400) 4400

dropout_3 (Dropout) (None, 400) 0

dense_5 (Dense) (None, 200) 80200

batch_normalization_2 (Batc (None, 200) 800

hNormalization)

dropout_4 (Dropout) (None, 200) 0

dense_6 (Dense) (None, 100) 20100

batch_normalization_3 (Batc (None, 100) 400

hNormalization)

dropout_5 (Dropout) (None, 100) 0

dense_7 (Dense) (None, 1) 101

=================================================================

Total params: 106,001

Trainable params: 105,401

Non-trainable params: 600

## Grid Search for hyperparameters of ANN model for selected feature for the prediction of MACE

[INFO] performing random search...

[INFO] best score is 0.95 using {'learnRate': 0.001, 'hiddenLayerTwo': 100, 'hiddenLayerThree': 50, 'hiddenLayerOne': 200, 'epochs': 15, 'dropout': 0.4, 'batch_size': 8, 'alfa': 0.03}

[INFO] evaluating the best model...

accuracy: 94.56%

Model: "sequential_2"

_________________________________________________________________

Layer (type) Output Shape Param #

=================================================================

dense_8 (Dense) (None, 200) 2000

dropout_6 (Dropout) (None, 200) 0

dense_9 (Dense) (None, 100) 20100

batch_normalization_4 (Batc (None, 100) 400

hNormalization)

dropout_7 (Dropout) (None, 100) 0

dense_10 (Dense) (None, 50) 5050

batch_normalization_5 (Batc (None, 50) 200

hNormalization)

dropout_8 (Dropout) (None, 50) 0

dense_11 (Dense) (None, 1) 51

=================================================================

Total params: 27,801

Trainable params: 27,501

Non-trainable params: 300
