# Supplementary material for: Prediction of major adverse cardiac events in the emergency department using an artificial neural network with a systematic grid search
Source: Int J Emerg Med. 2024 Jan 4;17:4. doi: 10.1186/s12245-023-00573-2 (PMC10768150; doi:10.1186/s12245-023-00573-2)
Supplement: Supplementary file 2 — Additional file 2: Table S2. Sensitivity & Specificity analysis of the reference and machine learning models in overall & selected training set. [file 12245_2023_573_MOESM2_ESM.docx]

**Table-S2:** **Sensitivity & Specificity analysis of the reference and machine learning models in overall & selected training set**

|  | **Overall Training** | | | **Selected features Training** | | |
| --- | --- | --- | --- | --- | --- | --- |
|  | **Accuracy** | **Sens.** | **Spec.** | **Acc.** | **Sens.** | **Spec.** |
|  | **(95% C.I)** | **(95% C.I)** | **(95% C.I)** | **(95% C.I)** | **(95% C.I)** | **(95% C.I)** |
| **In-hospital Mortality** |  |  |  |  |  |  |
| ANN Classifier | 93.70% | 95.80% | 93.60% | 84.05% | 92.85% | 92.39% |
|  | (93.5% to 93.9%) | (95.2% to 96.4%) | (93.4% to 93.8%) | (82.89% to 85.16%) | (92.66% to 93.04%) | (92.20% to 92.57%) |
| Random Forest Classifier | 93.30% | 88.80% | 93.60% | 86.99% | 92.79% | 92.50% |
|  | (93.1% to 93.5%) | (87.8% to 89.7%) | (93.4% to 93.7%) | (85.90% to 88.04%) | (92.60% to 92.98%) | (92.32% to 92.69%) |
| Logistic Regression | 92.50% | 77.70% | 93.50% | 82.59% | 92.74% | 92.21% |
|  | (92.3% to 92.6%) | (76.6% to 78.9%) | (93.3% to 93.7%) | (81.39% to 83.75%) | (92.55% to 92.93%) | (92.02% to 92.40%) |
| **Cardiac Arrest** |  |  |  |  |  |  |
| ANN Classifier | 97.20% | 95% | 97.30% | 88.5% | 96.4% | 96.2% |
|  | (97.1% to 97.3%) | (94.0% to 95.8%) | (97.2% to 97.4%) | (86.97% to 89.95%) | (96.23% to 96.49%) | (96.04% to 96.31%) |
| Random Forest Classifier | 97.10% | 98.80% | 97.10% | 89.48% | 96.42% | 96.26% |
|  | (97.07% to 97.3%) | (98.2% to 99.2%) | (97.0% to 97.2%) | (88.00% to 90.84%) | (96.29% to 96.55%) | (96.12% to 96.39%) |
| Logistic Regression | 95.63% | 70.83% | 96.40% | 65.50% | 96.37% | 95.37% |
|  | (95.4% to 95.7%) | (68.9% to 72.6%) | (96.3% to 96.5%) | (63.61% to 67.36%) | (96.24% to 96.51%) | (95.22% to 95.52%) |
| **Major Adverse Cardiac Events (MACE)** | | | | | | |
| ANN Classifier | 95.50% | 99.20% | 94.60% | 99.96% | 93.47% | 94.66% |
|  | (95.4% to 95.7%) | (99.1% to 99.4%) | (94.4% to 94.8%) | (99.91% to 99.98%) | (93.27% to 93.66%) | (94.50% to 94.81%) |
| Random Forest Classifier | 95.50% | 99.40% | 94.50% | 99.96% | 93.47% | 94.66% |
|  | (95.3% to 95.6%) | (99.2% to 99.5%) | (94.4% to 94.7%) | (99.91% to 99.98%) | (93.27% to 93.66%) | (94.50% to 94.81%) |
| Logistic Regression | 95.10% | 98.70% | 94.20% | 99.96% | 93.47% | 94.66% |
|  | (94.9% to 95.2%) | (98.5% to 98.9%) | (94.06% to 94.4%) | (99.91% to 99.98%) | (93.27% to 93.66%) | (94.50% to 94.81%) |
| *Acc. = accuracy, Sens. = sensitivity, Spec. = specificity* | | | | | | |
